# Associations between ultra-processed food and drink consumption and biomarkers of chronic low-grade inflammation: exploring the mediating role of adiposity

**DOI:** 10.1007/s00394-025-03666-1

**Published:** 2025-04-09

**Authors:** Seán R. Millar, Janas M. Harrington, Ivan J. Perry, Catherine M. Phillips

**Affiliations:** 1 Centre for Health and Diet Research, School of Public Health, University College Cork, Cork, Ireland; 2 School of Public Health, Physiotherapy and Sports Science, University College Dublin, Dublin 4, Ireland

**Keywords:** Ultra-processed foods, Ultra-processed drinks, Low-grade inflammation, Adiposity

## Abstract

**Purpose:**

Higher ultra-processed food and drink (UPFD) consumption has been linked with increased risk of non-communicable diseases. Low-grade systemic inflammation may partly underlie this relationship, yet limited research on UPFDs exists in this context. We examined UPFD associations with inflammatory biomarkers and explored whether relationships are mediated by adiposity.

**Methods:**

This was a cross-sectional study of 1,986 middle- to older-aged men and women. Using the NOVA classification, UPFD weight ratios were calculated for each participant. Correlation and multivariate-adjusted linear regression analyses were used to test UPFD intake associations with a wide range of inflammatory biomarkers. Mediation analyses explored whether relationships were independent or mediated by adiposity, defined by body mass index (BMI) or waist-height ratio (WHtR).

**Results:**

Significant direct effects between UPFD consumption and higher levels of interleukin 6, tumour necrosis factor-alpha, white blood cell counts and constituent neutrophils, basophils, and the neutrophil-to-lymphocyte ratio, were observed in models which controlled for a range of potential confounders, and which additionally adjusted for BMI or WHtR. Higher levels of adiposity were found to mediate relationships between UPFD intake and biomarkers, with the percentage of total effect mediated ranging from 12.7 to 70.1% for models including BMI, and 13.5 to 64.5% for models including WHtR.

**Conclusions:**

Consumption of UPFDs is associated with a less optimal inflammatory biomarker profile and the total effect of UPFD intake on biomarker concentrations is likely due both to higher levels of adiposity related to UPFD consumption and the pro-inflammatory potential of UPFD products.

**Supplementary Information:**

The online version contains supplementary material available at 10.1007/s00394-025-03666-1.

## Introduction

The rising consumption of ultra-processed foods and drinks (UPFDs) has become a public health concern in recent years. Changes in global food systems have led to UPFDs becoming readily available and dominating diet in many populations. However, UPFDs typically have high energy density and low nutritional value [1].

The NOVA classification enables food and drink items to be categorised into four groups based upon their level of processing [1, 2]. According to the NOVA classification, UPFDs are defined as formulations which contain little to no intact foods, as well as fats, salt, sugar, stabilisers, colourings, preservatives and emulsifiers added by manufacturers [1]. Since the initial publication of the NOVA system in 2009 [3], several large-scale cohort studies have demonstrated consumption of UPFDs to be related to a wide range of poor health outcomes [4]. Nevertheless, despite this global epidemiological evidence, biological mechanisms linking UPFDs with chronic non-communicable disease risk are still poorly understood [5].

Research over the last decade has shown low-grade systemic inflammation and raised immune activation to be associated with chronic conditions including type 2 diabetes, cardiovascular disease, neurodegenerative disease and many cancers [6–11]. The origins of chronic low-grade inflammation are thought to be multifactorial; continued exposure to different environmental and lifestyle factors are believed to result in the presence of immune cells such as lymphocytes, macrophages and pro-inflammatory cytokines in body tissue for long periods of time [12]. These may inhibit glucose uptake and alter lipid metabolism [13, 14].

Among the environmental and lifestyle factors that can promote chronic inflammation, increasing scientific evidence supports the role of diet [12, 15], and numerous studies have now demonstrated relationships between different dietary patterns and biomarkers of inflammation [15–19]. However, few have considered the effects of UPFDs on inflammatory markers and the limited available evidence is inconsistent [12]. It is also unclear as to whether UPFDs exert a direct effect on inflammatory biomarker levels or whether associations are fully mediated by higher levels of adiposity that are believed to be a consequence of UPFD consumption [20]. Moreover, the focus on inflammatory profiling in this context has been restricted to a narrow range of biomarkers.

When exploring associations between UPFDs and low-grade inflammation it is necessary to examine different biomarkers representing various aspects of cellular and organ sources of inflammation. This is important considering the complex inter-relationships between certain inflammatory biomarkers. Therefore, the aim of the present study was to assess relationships between UPFD consumption, classified using the NOVA system, and a broad range of inflammatory biomarkers including pro-inflammatory cytokines, adipocytokines, acute-phase response proteins, white blood cell counts (WBC) and WBC constituents. Using a random sample of 1,986 middle- to older-aged men and women we tested the hypothesis that higher intake of UPFDs would be associated with a more pro-inflammatory profile. We also explored whether this relationship was independent or mediated by adiposity.

## Materials and methods

### Study population and setting

The Cork and Kerry Diabetes and Heart Disease Study (Phase II– Mitchelstown Cohort) was a single-centre, cross-sectional study conducted between 2010 and 2011. Full details regarding the study design have been previously described [21]. In brief, the study population were patients that attended the Living Health Clinic in Mitchelstown, County Cork, Ireland. Participants were randomly selected from all registered middle- to older-aged (46–70 years) attending patients and a stratified sample was recruited. Of 3,807 potential participants, following the exclusion of duplicates, deaths and individuals who were incapable of consenting or attending appointment, 3,051 were invited to take part in the study. Of these, 2,047 (49% male) completed the questionnaire and physical examinations of the baseline assessment (response of 67%). Dietary data for the current analysis were available for 1,986 participants. A participant flow chart is shown in Supplementary Fig. 1. The sample was broadly similar to the local background population in terms of proportional age representation, marital status and education, representing a low risk of selection bias.

Ethics committee approval conforming to the Declaration of Helsinki was obtained from the Clinical Research Ethics Committee of University College Cork. A letter signed by the contact GP in the clinic was sent out to all selected participants with a reply slip indicating acceptance or refusal. All participants provided written consent to use their data for research purposes.

### Clinical procedures

Study participants attended the clinic in the morning after an overnight fast and blood samples were taken on arrival. Fasting glucose and glycated haemoglobin A_1c_ (HbA_1c_) concentrations were measured in fresh samples by Cork University Hospital Biochemistry Laboratory using standardised procedures. Glucose concentrations were determined using a glucose hexokinase assay (Olympus Life and Material Science Europa Ltd., Lismeehan, Co. Clare, Ireland) and HbA_1c_ levels were measured in the haematology laboratory on an automated high-pressure liquid chromatography instrument Tosoh G7 [Tosoh HLC-723 (G7), Tosoh Europe N.V, Tessenderlo, Belgium]. C-reactive protein (CRP), tumour necrosis factor-alpha (TNF-α), interleukin 6 (IL-6), adiponectin, leptin, resistin and plasminogen activator inhibitor-1 (PAI-1) were assessed using a biochip array system (Evidence Investigator; Randox Laboratories, UK). Complement component 3 (C3) was measured by immunoturbidimetric assay (RX Daytona; Randox Laboratories). White blood cell count (WBC), neutrophil, lymphocyte, monocyte, eosinophil and basophil concentrations were determined by flow cytometry technology as part of a full blood count. The neutrophil-to-lymphocyte ratio (NLR) was calculated as neutrophils divided by lymphocytes.

Clinical measurements were performed by trained researchers with reference to a standard operating procedures manual. Height was measured with a portable Seca Leicester height/length stadiometer (Seca, Birmingham, UK) and weight was measured using a portable electronic Tanita WB-100MA weighing scale (Tanita Corp, IL, USA). The weighing scale was placed on a firm flat surface and was calibrated weekly. Body mass index (BMI = weight(kg)/height(m)^2^) was calculated from measured weight and height. Waist circumference was measured between the lowest rib and iliac crest on bare skin. Participants were instructed to breathe in, and then out, and to hold their breath while measurement was made to the nearest 0.1 cm using a Seca 200 measuring tape. The mean of two independent readings was used in analysis. Height was divided into waist circumference measurements to derive the waist-height ratio (WHtR). We used two adiposity indices in our analyses for cross-validation as BMI is a measure of general adiposity while WHtR is a measure of central adiposity. Although BMI is more frequently used in a clinical setting to assess body composition, it has been criticised as being an inaccurate measure of body fat [22]. In addition, neither BMI or WHtR require sex-specific thresholds to be used in analyses [23].

### Data collection and dietary assessment

A general health and lifestyle questionnaire (GHLQ) assessed demographic variables, lifestyle behaviours and morbidity. Information on age, sex, education, use of prescription medications, smoking status, alcohol use and morbidity was provided by participants. Physical activity levels were measured using the validated International Physical Activity Questionnaire (IPAQ) [24].

Diet was evaluated using a version of the self-completed European Prospective Investigation into Cancer and Nutrition (EPIC) Food Frequency Questionnaire (FFQ) [25], which has been validated extensively in several populations [26]. This FFQ was modified to reflect the Irish diet by the National Nutrition Surveillance Centre in Ireland and was validated for use in the Irish population using food diaries and a protein biomarker in a volunteer sample [27] and incorporated into the SLÁN Irish National Surveys of Lifestyle Attitudes and Nutrition 1998, 2002 and 2007 [28–30]. Data on food frequency consumption during the past 12 months were gathered. Using a tailored computer programme (FFQ Software Version 1.0; developed by the National Nutrition Surveillance Centre, School of Public Health, Physiotherapy and Sports Science, University College Dublin, Belfield, Dublin 4, Ireland), the daily intake of energy and nutrients was computed from the FFQ data by linking food equivalents in McCance and Widdowson Food Tables to food frequency selections [31].

### NOVA classification

The NOVA classification divides foods into four broad categories based on their level of processing [1]. Specific NOVA food groups, definitions and representative foods are shown in Supplementary Table 1. The 149 foods and beverages included in the FFQ were categorised based on their level of processing according to the NOVA system. For the purpose of the current analysis, the fourth NOVA group, ultra-processed foods and ultra-processed drinks, was examined. A list of foods and drinks categorised according to the NOVA classification is presented in Supplementary Table 2. We summed the amount consumed (grams/day and kilocalories/day) of each food and drink item from the fourth NOVA group. In total, 67 items comprising 59 foods and 8 drinks were classified as UPFDs. The UPFD weight ratio was calculated by dividing the weight (in grams) of UPFDs consumed by the weight of all food and drinks consumed. We used the UPFD weight ratio in our main analyses to express the quantity of UPFDs consumed because some foods and beverages do not provide energy. For sensitivity analyses we also calculated the percentage contribution of UPFDs to total energy intake (UPFD percentage energy intake) by dividing participants’ UPFD energy intake by total energy intake then multiplying by 100.

### Covariates

Education was defined as ‘secondary or higher’ or ‘primary level only’. Smoking status was defined as ‘non-smoker’ and ‘current smoker’. Alcohol consumption was measured in units of alcohol consumed on a weekly basis and was categorised into the following levels: (i) non-drinker, i.e. <1 drink per week; (ii) moderate drinker, i.e. between 1 and 14 drinks per week; and (iii) heavy drinker, i.e. >14 drinks per week. Moderate drinker was defined on the basis of previous work from the EPIC in the United Kingdom by Khaw et al. [32]. For the current analysis, these were then re-categorised as ‘moderate/non-drinker’ or ‘heavy drinker’. Physical activity was categorised as high, moderate or low levels of activity using the IPAQ. This was then recoded as a dichotomous variable: ‘moderate/high’ or ‘low’ level physical activity.

Type 2 diabetes was determined as a fasting glucose level *≥* 7.0 mmol/l or HbA_1c_ level *≥* 6.5% (*≥* 48 mmol/mol) [33] or by self-reported diagnosis. The presence of cardiovascular disease was obtained by asking study participants if they had been diagnosed with any one of the following seven conditions: Heart Attack (including coronary thrombosis or myocardial infarction), Heart Failure, Angina, Aortic Aneurysm, Hardening of the Arteries, Stroke or any other Heart Trouble. Subjects who indicated a diagnosis of any one of these conditions were classified as having cardiovascular disease. Cancer was defined as a binary (yes/no) variable as determined from participants’ self-reported responses to the GHLQ.

### Statistical analysis

Descriptive characteristics were examined according to UPFD weight ratio quartiles. Categorical features are presented as numbers (n) and percentages (%) and continuous variables are shown as a mean, plus or minus one standard deviation (± SD), or a median and interquartile range (IQR) for skewed data. Differences between quartiles 4 and 1 were analysed using a Pearson’s chi-square test, Student’s *t*-test or a Mann Whitney U. To assess whether UPFD consumption and total food intake were proportional, we used a scatterplot and a restricted cubic spline model with five knots to examine the relationship between total UPFD intake (log grams) and total food intake (log grams) in order to identify any non-linear patterns.

Correlations between UPFD intake and inflammatory biomarkers were examined using Spearman’s rank-order correlation. Participants’ UPFD weight ratios and UPFD percentage energy intake scores were standardised by subtracting the sample mean and dividing by the standard deviation. Biomarker data were log-transformed and linear regression analyses were performed. Three models were run to test relationships between UPFD weight ratios (independent variable) and inflammatory biomarkers (dependent variable). Model 1 was age and sex-adjusted while Model 2 additionally adjusted for total energy intake. A third model also adjusted for education, lifestyle behaviours (smoking, alcohol use, physical activity), anti-inflammatory medication use, type 2 diabetes, cardiovascular disease history and cancer. Additional sensitivity linear regression analyses were conducted: one which excluded participants with prevalent diseases (type 2 diabetes, cardiovascular diseases and cancer; *n* = 407) and another which used UPFD percentage energy intake as the independent variable. Data analysis was conducted using Stata SE Version 13 (Stata Corporation, College Station, TX, USA) for Windows. For all analyses, a *p* value (two-tailed) of less than 0.05 was considered to indicate statistical significance.

To further explore whether relationships between UPFD (weight ratio) intake and inflammatory biomarkers are mediated by adiposity, defined by BMI or WHtR, we conducted mediation analyses. For any biomarker that demonstrated a significant relationship with UPFD intake in Model 3, we calculated direct and indirect effects with 95% confidence intervals determined from 5000 bootstrap samples using the PROCESS macro [34] in IBM SPSS Statistics 25 (IBM Corp., Armonk, NY, USA). We also performed the Sobel test of mediation using the sgmediation2 command in Stata [35]. Evidence of mediation was considered on the basis of an indirect effect with confidence intervals that did not include the null value and a Sobel test *p* value less than 0.05. For significant indirect effects, the percentage of total effect mediated was also calculated. All mediation analyses were adjusted for age, sex, total energy intake, education, lifestyle behaviours (smoking, alcohol use, physical activity), anti-inflammatory medication use, type 2 diabetes, cardiovascular disease history and cancer. The linearity and homoscedasticity assumptions of mediation analysis were assessed using the multiple regression standardised predicted and residual values.

To account for the multiple testing performed in fully adjusted regression models, and in mediation analyses, we also calculated false discovery rate (FDR) adjusted *p* values using the Benjamini and Hochberg correction procedure for multiple testing with a FDR of 0.05 [36]. For any inflammatory biomarker that demonstrated a significant direct effect with UPFD consumption, we additionally tested correlations with nine mutually exclusive UPFD NOVA subgroups as described in Supplementary Table 2. There were low levels of missing data in our sample. Missing data were therefore considered ignorable and missing at random.

## Results

### Descriptive characteristics

Table 1 shows characteristics of the sample according to UPFD weight ratio quartiles. Among the study population, UPFDs accounted for 42.3% of average total daily energy intake, with ultra-processed foods and ultra-processed drinks accounting for 41.0% and 1.3% of average total daily energy intake, respectively. Participants who consumed greater amounts UPFDs (quartile 4 compared to quartile 1) were younger, more likely to be male and to have been educated to a primary level only. Participants with higher UPFD intake were also more likely to have low levels of physical activity and a higher BMI and WHtR. A greater percentage of participants with lower UPFD intake were heavy drinkers of alcohol. Significant differences in inflammatory biomarker levels between quartiles 4 and 1 were observed for C3, CRP, IL-6, TNF-α, adiponectin, leptin, resistin, WBCs and WBC constituents, and the NLR.

**Table 1:** Descriptive characteristics of the study sample according to UPFD (weight ratio) intake quartiles

Variable	Total	UPFD weight ratio quartiles				
	(*n* = 1986)	Q1	Q2	Q3	Q4	*p*
**Dietary intake**						
Total food intake, grams/day (mean ± SD)	2569.6 ± 907.5	2645.4 ± 1020.3	2623.5 ± 825.1	2571.3 ± 838.7	2438.6 ± 921.1	0.001
Total UPFD intake, grams/day (mean ± SD)	444.0 ± 256.9	220.9 ± 99.6	368.0 ± 120.9	491.1 ± 167.1	695.4 ± 301.0	< 0.001
Total energy intake, kilocalories/day (mean ± SD)	2038.9 ± 809.9	1699.3 ± 701.2	1964.7 ± 692.4	2130.0 ± 738.7	2361.2 ± 936.4	< 0.001
UPFD percentage energy intake (mean ± SD)	42.3 ± 13.5	27.7 ± 8.9	39.5 ± 8.2	47.0 ± 8.6	54.8 ± 10.5	< 0.001
**General**						
Age in years (median, IQR)	59 (54.5, 64.0)	60.0 (55.0, 64.0)	59.0 (54.0, 64.0)	59.0 (54.0, 63.0)	58.0 (54.0, 63.0)	0.006
Male (n, %)	974 (49.0)	218 (44.0)	234 (47.1)	254 (51.2)	268 (53.9)	0.002
Primary education only (n, %)	522 (26.3)	107 (21.6)	123 (24.7)	141 (28.4)	151 (30.4)	0.002
Current smoker (n, %)	282 (14.2)	70 (14.1)	75 (15.1)	65 (13.1)	72 (14.5)	0.866
Heavy drinker (n, %)	191 (9.6)	78 (15.7)	42 (8.5)	39 (7.9)	32 (6.4)	< 0.001
Low-level physical activity (n, %)	905 (45.6)	210 (42.3)	214 (43.1)	231 (46.6)	250 (50.3)	0.012
BMI, kg/m^2^ (mean ± SD)	28.5 ± 21.9	28.0 ± 4.6	28.1 ± 4.6	28.7 ± 4.7	29.4 ± 4.7	< 0.001
WHtR (mean ± SD)	0.58 ± 0.07	0.57 ± 0.07	0.58 ± 0.07	0.59 ± 0.07	0.59 ± 0.07	< 0.001
Anti-inflammatory medication use (n, %)	330 (16.9)	77 (15.7)	74 (15.1)	88 (18.3)	91 (18.6)	0.23
Type 2 diabetes (n, %)	178 (9.0)	35 (7.1)	39 (7.8)	54 (10.9)	50 (10.1)	0.091
Cardiovascular disease (n, %)	206 (10.4)	51 (10.3)	53 (10.7)	50 (10.1)	52 (10.5)	0.926
Cancer (n, %)	79 (4.0)	18 (3.6)	22 (4.4)	18 (3.6)	21 (4.2)	0.629
**Inflammatory biomarkers**						
C3, mg/dl (median, IQR)	134.1 (120.4, 149.4)	131.9 (117.2, 147.3)	133.3 (120.8, 147.8)	133.4 (120.9, 149.4)	136.6 (122.8, 152.4)	0.001
CRP, ng/ml (median, IQR)	1.4 (1.0, 2.3)	1.2 (0.9, 1.9)	1.4 (1.0, 2.3)	1.4 (1.0, 2.3)	1.5 (1.0, 2.5)	< 0.001
IL-6, pg/ml (median, IQR)	1.8 (1.2, 2.9)	1.6 (1.13, 2.6)	1.7 (1.2, 2.9)	1.8 (1.2, 2.8)	2.0 (1.3, 3.1)	< 0.001
TNF-α, pg/ml (median, IQR)	6.0 (4.9, 7.3)	5.8 (4.7, 7.0)	5.9 (4.8, 7.2)	6.1 (5.0, 7.5)	6.2 (5.1, 7.5)	< 0.001
Adiponectin, ng/ml (median, IQR)	4.7 (2.9, 7.5)	5.2 (3.0, 8.1)	4.9 (3.0, 7.4)	4.4 (2.9, 7.5)	4.4 (2.8, 6.9)	0.001
Leptin, ng/ml (median, IQR)	2.0 (1.1, 3.1)	1.7 (1.0, 3.0)	2.0 (1.2, 3.1)	1.8 (1.1, 2.8)	2.2 (1.2, 3.5)	0.003
Resistin, ng/ml (median, IQR)	5.1 (3.9, 6.7)	5.0 (3.9, 6.5)	5.0 (3.9, 6.8)	4.9 (4.0, 6.6)	5.3 (4.0, 6.9)	0.036
PAI-1, ng/ml (median, IQR)	26.0 (19.1, 34.0)	25.1 (18.8, 33.3)	26.4 (18.5, 34.1)	26.8 (19.7, 34.2)	26.2 (18.7, 34.1)	0.475
WBC, 10^9^/l (median, IQR)	5.7 (4.8, 6.8)	5.5 (4.7, 6.5)	5.7 (4.7, 6.7)	5.8 (4.9, 6.8)	5.9 (4.9, 7.2)	< 0.001
Neutrophils, 10⁹/l (median, IQR)	3.1 (2.5, 3.9)	2.9 (2.4, 3.7)	3.1 (2.5, 3.9)	3.3 (2.5, 4.0)	3.3 (2.6, 4.1)	< 0.001
Lymphocytes, 10⁹/l (median, IQR)	1.7 (1.4, 2.1)	1.7 (1.5, 2.2)	1.7 (1.4, 2.1)	1.8 (1.4, 2.1)	1.8 (1.4, 2.2)	0.825
NLR (median, IQR)	1.8 (1.4, 2.3)	1.6 (1.3, 2.1)	1.8 (1.4, 2.3)	1.8 (1.4, 2.4)	1.8 (1.5, 2.4)	< 0.001
Monocytes, 10⁹/l (median, IQR)	0.50 (0.4, 0.6)	0.48 (0.4, 0.6)	0.49 (0.4, 0.6)	0.51 (0.4, 0.6)	0.50 (0.4, 0.6)	0.082
Eosinophils, 10⁹/l (median, IQR)	0.17 (0.11, 0.26)	0.17 (0.1, 0.3)	0.17 (0.1, 0.3)	0.17 (0.1, 0.3)	0.18 (0.1, 0.3)	0.059
Basophils, 10⁹/l (median, IQR)	0.030 (0.020, 0.040)	0.031 (0.02, 0.04)	0.032 (0.02, 0.04)	0.032 (0.02, 0.04)	0.033 (0.02, 0.04)	0.053

Abbreviations BMI: body mass index; C3: complement component 3; CRP: c-reactive protein; IL-6: interleukin 6; NLR: neutrophil-to-lymphocyte ratio; PAI-1: plasminogen activator inhibitor 1; TNF-α: tumour necrosis factor-alpha; UPFD: ultra-processed food and drink; WBC: white blood cell count; WHtR: waist-height ratio. *p* values determined from a chi-square test, *t*-test or Mann Whitney U and compare Q4 to Q1

In Supplementary Figs. 2 and 3, a scatterplot demonstrated a linear relationship between total UPFD intake and total food intake, with no evidence of non-linearity in a restricted cubic spline model (Wald test for linear term: *p* < 0.001). With regard to dietary composition, dietary macronutrient profiles were noticeably different across UPFD weight ratio quartiles (Supplementary Table 3). Participants with higher UPFD intake demonstrated higher consumption of saturated fatty acids, polyunsaturated fatty acids, monounsaturated fatty acids, carbohydrates, protein and sugar, and lower consumption of alcohol. Study participants with higher UPFD intake were also observed to consume greater amounts of bread and cereals, lower amounts of fruit and vegetables and greater amounts of high fat/sugar foods and drink products according to daily servings based on food pyramid recommendations.

### Correlation analysis

In correlation analyses (Table 2), weak but significant positive correlations between UPFD intake and biomarkers were observed for concentrations of C3, CRP, IL-6, TNF-α, leptin, resistin, WBCs, neutrophils, the NLR, monocytes and eosinophils. Adiponectin levels were inversely correlated with UPFD intake. Relationships between UPFD consumption and inflammatory biomarkers were stronger for WBCs and TNF-α, with the NLR and neutrophils showing the highest correlative strengths.

**Table 2:** Spearman correlation coefficients between UPFD (weight ratio) intake and inflammatory biomarkers

Biomarker	Correlation coefficients	*p*
C3, mg/dl	0.081	**< 0.001**
CRP, ng/ml	0.091	**< 0.001**
IL-6, pg/ml	0.093	**< 0.001**
TNF-α, pg/ml	0.104	**< 0.001**
Adiponectin, ng/ml	-0.097	**< 0.001**
Leptin, ng/ml	0.057	**0.012**
Resistin, ng/ml	0.054	**0.018**
PAI-1, ng/ml	0.021	0.347
WBC, 10^9^/l	0.100	**< 0.001**
Neutrophils, 10⁹/l	0.128	**< 0.001**
Lymphocytes, 10⁹/l	-0.002	0.945
NLR	0.108	**< 0.001**
Monocytes, 10⁹/l	0.052	**0.021**
Eosinophils, 10⁹/l	0.047	**0.040**
Basophils, 10⁹/l	0.039	0.087

Abbreviations C3: complement component 3; CRP: c-reactive protein; IL-6: interleukin 6; NLR: neutrophil-to-lymphocyte ratio; PAI-1: plasminogen activator inhibitor 1; TNF-α: tumour necrosis factor-alpha; UPFD: ultra-processed food and drink; WBC: white blood cell count. Values are presented as Spearman correlation coefficients between UPFD weight ratios and inflammatory biomarkers among the Mitchelstown Cohort (*n* = 1986). Significant *p* shown in **bold**

### Linear regression

The results of linear regression analyses which investigated associations between UPFD consumption and inflammatory biomarkers are shown in Table 3. In models which adjusted for age, sex and total energy intake, significant relationships were observed between UPFD weight ratios and 12 of the 14 examined biomarkers and the NLR. Upon full adjustment, UPFD intake associations with higher concentrations of C3 (β = 0.015, *p* = 0.02), CRP (β = 0.048, *p* = 0.006), IL-6 (β = 0.065, *p* < 0.001), TNF-α (β = 0.025, *p* = 0.004), leptin (β = 0.059, *p* = 0.006), resistin (β = 0.024, *p* = 0.023), WBCs (β = 0.021, *p* = 0.001), neutrophils (β = 0.035, *p* < 0.001), monocytes (β = 0.017, *p* = 0.025), eosinophils (β = 0.033, *p* = 0.029), basophils (β = 0.032, *p* = 0.021) and the NLR (β = 0.035, *p* < 0.001) remained. These relationships withstood a Benjamini and Hochberg multiple hypothesis correction.

**Table 3:** Linear regression analysis of the associations between UPFD (weight ratio) intake and inflammatory biomarkers

Biomarker	Model 1			Model 2			Model 3			
	β	95% CI	*p*	β	95% CI	*p*	β	95% CI	*p*	*p* (FDR)
C3	0.018	0.006, 0.029	**0.003**	0.019	0.007, 0.031	**0.002**	0.015	0.002, 0.027	**0.02**	**0.034**
CRP	0.049	0.017, 0.081	**0.002**	0.058	0.025, 0.091	**0.001**	0.048	0.014, 0.082	**0.006**	**0.013**
IL-6	0.066	0.034, 0.099	**< 0.001**	0.076	0.042, 0.110	**< 0.001**	0.065	0.030, 0.099	**< 0.001**	**0.001**
TNF-α	0.030	0.015, 0.046	**< 0.001**	0.030	0.014, 0.046	**< 0.001**	0.025	0.008, 0.042	**0.004**	**0.012**
Adiponectin	-0.033	-0.060, -0.006	**0.018**	-0.034	-0.063, -0.006	**0.018**	-0.021	-0.051, 0.008	0.149	0.172
Leptin	0.059	0.019, 0.098	**0.004**	0.068	0.027, 0.109	**0.001**	0.059	0.017, 0.102	**0.006**	**0.013**
Resistin	0.027	0.007, 0.046	**0.007**	0.031	0.011, 0.052	**0.002**	0.024	0.003, 0.045	**0.023**	**0.034**
PAI-1	-0.008	-0.030, 0.014	0.474	-0.007	-0.031, 0.016	0.535	-0.010	-0.035, 0.014	0.399	0.428
WBC	0.023	0.010, 0.035	**< 0.001**	0.030	0.017, 0.043	**< 0.001**	0.021	0.009, 0.034	**0.001**	**0.004**
Neutrophils	0.036	0.021, 0.051	**< 0.001**	0.046	0.030, 0.061	**< 0.001**	0.035	0.019, 0.051	**< 0.001**	**0.001**
Lymphocytes	0.004	-0.011, 0.018	0.634	0.007	-0.009, 0.022	0.406	0.000	-0.015, 0.016	0.95	0.95
NLR	0.032	0.015, 0.050	**< 0.001**	0.039	0.021, 0.058	**< 0.001**	0.035	0.015, 0.054	**< 0.001**	**0.001**
Monocytes	0.013	-0.001, 0.027	0.074	0.020	0.005, 0.035	**0.008**	0.017	0.002, 0.032	**0.025**	**0.034**
Eosinophils	0.030	0.002, 0.058	**0.037**	0.042	0.013, 0.071	**0.005**	0.033	0.004, 0.063	**0.029**	**0.036**
Basophils	0.029	0.004, 0.054	**0.024**	0.035	0.008, 0.061	**0.01**	0.032	0.005, 0.059	**0.021**	**0.034**

Abbreviations C3: complement component 3; CRP: c-reactive protein; FDR: false discovery rate; IL-6: interleukin 6; NLR: neutrophil-to-lymphocyte ratio; PAI-1: plasminogen activator inhibitor 1; TNF-α: tumour necrosis factor-alpha; UPFD: ultra-processed food and drink; WBC: white blood cell count

Model 1: adjusted for age (in years, continuous) and sex (binary)

Model 2: adjusted for age (in years, continuous), sex (binary) and total energy intake (kilocalories, continuous)

Model 3: adjusted for age (in years, continuous), sex (binary), total energy intake (kilocalories, continuous), education (binary), smoking (binary), alcohol use (binary), physical activity (binary), anti-inflammatory medication use (binary), type 2 diabetes (binary), cardiovascular disease (binary) and cancer (binary)

Beta (β) coefficients and 95% confidence intervals (CIs) are shown. Significant *p* shown in **bold**

In sensitivity analyses which excluded study participants with prevalent diseases (Supplementary Table 4), similar magnitudes and directions of association were observed, with relationships between UPFD weight ratios and concentrations of C3, CRP, IL-6, TNF-α, leptin, resistin, WBCs, neutrophils and the NLR withstanding a multiple hypothesis correction. However, associations between standardised UPFD percentage energy intake scores and a majority of biomarkers were noticeably weaker (Supplementary Table 5), with only resistin, neutrophils and the NLR demonstrating significant relationships after correcting for multiple testing.

### Mediation

In mediation analyses (Tables 4 and 5) there was evidence that higher levels of adiposity (defined by BMI or WHtR) mediate relationships between UPFD consumption and increased concentrations of C3, CRP, IL-6, TNF-α, leptin, resistin, WBCs, neutrophils and monocytes, as indicated by significant indirect effects (confidence intervals that did not include the null value and Sobel test *p* values less than 0.05). These indirect effects withstood a multiple hypothesis correction. The percentage of total effect mediated was similar for both measures of general and central adiposity, but varied according to the biomarker examined, ranging from 12.7 to 70.1% for models including BMI, and 13.5 to 64.5% for models including WHtR.

**Table 4:** Mediating effect of BMI on the associations between UPFD (weight ratio) intake and inflammatory biomarkers

Biomarker	Direct effect of UPFD intake on biomarker				Indirect effect through BMI		Sobel test of mediation			Conclusion	% of total effect mediated
	β	95% CI	*p*	*p* (FDR)	β	95% CI	**z**	*p*	*p* (FDR)		
C3	0.010	-0.003, 0.022	0.117	0.128	0.005	0.002, 0.009	3.150	**0.002**	**0.006**	**Mediation**	34.3
CRP	0.028	-0.004, 0.061	0.089	0.107	0.020	0.008, 0.033	3.379	**0.001**	**0.004**	**Mediation**	41.3
IL-6	0.051	0.017, 0.085	**0.003**	**0.012**	0.014	0.005, 0.023	3.255	**0.001**	**0.004**	**Mediation**	20.9
TNF-α	0.022	0.005, 0.039	**0.012**	**0.029**	0.003	0.001, 0.006	2.761	**0.006**	**0.009**	**Mediation**	12.7
Leptin	0.018	-0.018, 0.053	0.327	0.327	0.042	0.017, 0.067	3.458	**0.001**	**0.004**	**Mediation**	70.1
Resistin	0.021	-0.000, 0.042	0.054	0.072	0.004	0.001, 0.007	2.747	**0.006**	**0.009**	**Mediation**	15.7
WBC	0.018	0.005, 0.030	**0.005**	**0.015**	0.004	0.001, 0.006	2.912	**0.004**	**0.008**	**Mediation**	20.4
Neutrophils	0.030	0.015, 0.046	**< 0.001**	**0.001**	0.005	0.002, 0.008	2.902	**0.004**	**0.008**	**Mediation**	12.9
NLR	0.033	0.014, 0.053	**0.001**	**0.006**	0.001	-0.000, 0.003	1.447	0.148	0.161	No mediation	-
Monocytes	0.015	-0.000, 0.029	0.053	0.072	0.002	0.001, 0.004	2.508	**0.012**	**0.016**	**Mediation**	13.5
Eosinophils	0.031	0.001, 0.061	**0.044**	0.072	0.024	0.000, 0.005	1.771	0.077	0.092	No mediation	-
Basophils	0.032	0.004, 0.059	**0.023**	**0.046**	0.000	-0.002, 0.003	0.297	0.767	0.767	No mediation	-

Abbreviations BMI: body mass index; C3: complement component 3; CRP: c-reactive protein; FDR: false discovery rate; IL-6: interleukin 6; NLR: neutrophil-to-lymphocyte ratio; PAI-1: plasminogen activator inhibitor 1; TNF-α: tumour necrosis factor-alpha; UPFD: ultra-processed food and drink; WBC: white blood cell count

All models adjusted for age (in years, continuous), sex (binary), total energy intake (kilocalories, continuous), education (binary), smoking (binary), alcohol use (binary), physical activity (binary), anti-inflammatory medication use (binary), type 2 diabetes (binary), cardiovascular disease (binary) and cancer (binary)

Beta (β) coefficients and 95% confidence intervals (CI) determined from 5000 bootstrap samples are shown. Significant *p* shown in **bold**

**Table 5:** Mediating effect of WHtR on the associations between UPFD (weight ratio) intake and inflammatory biomarkers

Biomarker	Direct effect of UPFD intake on biomarker				Indirect effect through WHtR		Sobel test of mediation			Conclusion	% of total effect mediated
	β	95% CI	*p*	*p* (FDR)	β	95% CI	**z**	*p*	*p* (FDR)		
C3	0.010	-0.003, 0.022	0.128	0.14	0.005	0.002, 0.009	3.283	**0.001**	**0.003**	**Mediation**	36.2
CRP	0.029	-0.004, 0.061	0.086	0.103	0.019	0.008, 0.031	3.592	**< 0.001**	**0.001**	**Mediation**	40.4
IL-6	0.051	0.017, 0.085	**0.003**	**0.012**	0.014	0.006, 0.023	3.381	**0.001**	**0.003**	**Mediation**	21.8
TNF-α	0.021	0.004, 0.038	**0.014**	**0.034**	0.004	0.001, 0.006	2.942	**0.003**	**0.005**	**Mediation**	14.7
Leptin	0.021	-0.016, 0.059	0.267	0.267	0.038	0.018, 0.060	3.614	**< 0.001**	**0.001**	**Mediation**	64.5
Resistin	0.021	0.000, 0.042	**0.049**	0.074	0.003	0.001, 0.006	2.636	**0.008**	**0.011**	**Mediation**	14.0
WBC	0.017	0.005, 0.029	**0.006**	**0.018**	0.004	0.002, 0.007	3.084	**0.002**	**0.004**	**Mediation**	18.9
Neutrophils	0.030	0.015, 0.046	**< 0.001**	**0.001**	0.005	0.002, 0.008	3.027	**0.002**	**0.004**	**Mediation**	13.5
NLR	0.034	0.014, 0.053	**0.001**	**0.006**	0.001	-0.003, 0.003	1.310	0.19	0.221	No mediation	-
Monocytes	0.014	-0.000, 0.029	0.056	0.075	0.003	0.001, 0.005	2.636	**0.008**	**0.011**	**Mediation**	14.9
Eosinophils	0.032	0.002, 0.062	**0.039**	0.067	0.002	-0.000, 0.004	1.272	0.203	0.221	No mediation	-
Basophils	0.032	0.004, 0.059	**0.023**	**0.046**	0.000	-0.002, 0.002	0.175	0.861	0.861	No mediation	-

Abbreviations C3: complement component 3; CRP: c-reactive protein; FDR: false discovery rate; IL-6: interleukin 6; NLR: neutrophil-to-lymphocyte ratio; PAI-1: plasminogen activator inhibitor 1; TNF-α: tumour necrosis factor-alpha; UPFD: ultra-processed food and drink; WBC: white blood cell count; WHtR: waist-height ratio

All models adjusted for age (in years, continuous), sex (binary), total energy intake (kilocalories, continuous), education (binary), smoking (binary), alcohol use (binary), physical activity (binary), anti-inflammatory medication use (binary), type 2 diabetes (binary), cardiovascular disease (binary) and cancer (binary)

Beta (β) coefficients and 95% confidence intervals (CI) determined from 5000 bootstrap samples are shown. Significant *p* shown in **bold**

After correcting for multiple testing, significant direct effects between UPFD weight ratios and higher inflammatory biomarker concentrations were observed for IL-6, TNF-α, WBCs, neutrophils, basophils and the NLR in models which adjusted for either BMI or WHtR. Additional sensitivity analyses which explored UPFD NOVA subgroup correlations with these biomarkers (Supplementary Table 6) suggested ultra-processed drinks to be the main drivers of direct relationships between UPFD consumption and pro-inflammatory biomarker concentrations. No associations were observed with the ultra-processed cereal, dairy products/fats, potatoes/pasta and vegetable subgroups.

## Discussion

In this study of 1,986 middle- to older-aged men and women we examined relationships between UPFD consumption, defined using the NOVA classification, and a wide range of inflammatory biomarkers representing various aspects of cellular and organ sources of inflammation. In analyses which adjusted for age, sex, total energy intake, education, lifestyle behaviours, anti-inflammatory medication use and morbidity, significant positive associations between UPFD weight ratios and concentrations of C3, CRP, IL-6, TNF-α, leptin, resistin, WBCs and WBC constituents, and the NLR, were observed. These findings were robust to adjustment for multiple testing. In models which additionally included either BMI or WHtR, higher levels of adiposity were found to partially mediate relationships between UPFD consumption and inflammatory biomarker concentrations.

To date, only a small number of studies have examined the relationship between UPFDs and systemic inflammation and these have been restricted to a narrow range of biomarkers [37–40]. Results have been inconclusive, with some studies suggesting UPFD/inflammatory biomarker associations to be fully mediated by adiposity [12, 40]. Findings from our research demonstrate independent associations between UPFD consumption and inflammatory biomarker concentrations, with significant direct effects being observed between UPFD weight ratios and higher levels of IL-6, TNF-α, WBCs, neutrophils, basophils, and the NLR, in models which controlled for a range of potential confounders, and which additionally adjusted for adiposity defined using two separate measures. Consequently, as research has shown low-grade systemic inflammation and raised immune activation to be associated with a range of conditions [7, 8, 10], these results suggest a potential mechanism linking UPFD intake with increased chronic disease risk.

The factors which underlie observed direct effects between UPFD consumption and higher circulating inflammatory biomarker concentrations may include the food ingredients in UPFD products [12, 41], as our study and others have shown that the nutritional quality of diets with large amounts of UPFDs are more energy dense and contain more added sugars, refined carbohydrates and saturated fats [4]. UPFDs also typically displace minimally processed, nutritious foods that are the basis of a healthy and balanced diet [12, 41]. In particular, studies have shown that individuals who consume more UPFD products have a lower intake of fruit and vegetables [12, 42], and these findings were also observed in our research. Fruit and vegetables are believed to have anti-inflammatory effects due to the presence of numerous phytocompounds [12]. Nutrients such as vitamins C, D, E, selenium and carotenoids are antioxidants that act to reduce evolution of reactive species that can initiate chronic disease development through inflammation. Consuming fruits and vegetables that contain these and other nutrients may provide anti-inflammatory benefits [19]. Reduced fruit and vegetable consumption will also result in lower dietary fibre intake [12], and fibre and antioxidants may further exert a positive effect on inflammatory biomarker levels through maintaining homeostasis of the gut microbiota [15].

Sensitivity analyses suggested ultra-processed drinks to be the main drivers of the direct relationship between UPFD consumption and low-grade inflammation in this sample of middle- to older-aged adults. Sugary drinks, sweetened juices and energy drinks are known to be major contributors to excessive sugar intake and empty calories in modern diets. These beverages often contain high levels of simple sugars, in the form of either sucrose or high-fructose syrup, which may raise blood glucose markedly and rapidly [43]. This postprandial increase in glucose levels may in turn cause an increase in insulin levels, which promote a pro-inflammatory state [12, 44]. In a systematic review and meta-analyses of 1,211,470 participants, Li et al. found that greater sugar-sweetened beverage consumption was associated with a higher risk of cardiovascular disease mortality (HR = 1.20; 95% CI: 1.05, 1.38, *p* < 0.001) and all-cause mortality (HR = 1.12; 95% CI: 1.06, 1.19, *p* < 0.001) [45]. Notably, in a study of 38,261 subjects, Vellinga et al. found the association between UPFD intake and all-cause mortality to be predominantly driven by ultra-processed drink consumption [46]. It should be recognised, however, that the NOVA ultra-processed drinks subgroup includes various liquids, including alcoholic beverages and artificially sweetened beverages, which contain little to no calories or nutrients.

Notably, our study found associations between UPFD percentage energy intake scores and inflammatory biomarkers to be less consistent, and weaker, when compared to those observed using UPFD weight ratios, with only resistin, neutrophils and the NLR demonstrating significant relationships with UPFD percentage energy intake after correcting for multiple testing. These findings were surprising, as previous research by our group (using the same data) found both measures of UPFD intake to demonstrate the same relationships with a range of lipoprotein subclass biomarkers, with similar magnitudes of association [47]. Most investigators to date have only examined UPFD intake in the context of percentage energy intake. However, this approach has been criticised, as it does not take into account UPFDs that do not provide energy (artificially sweetened beverages for example) and non-nutritional factors associated with food processing such as neo-formed contaminants, food additives and alterations to the structure of raw foods [48]. It should be noted that in their study of 21,315 subjects, Mignogna et al. found UPFD associations with low-grade inflammation to be only partially explained by nutritional factors [49]. These findings support the hypothesis that chronic disease risk resulting from greater UPFD intake may arise not only from specific food items and nutrients (or a lack of) but also from additional, still not entirely understood, non-nutritional factors related to food processing [50].

Importantly, our research shows that the total effect of UPFD intake on inflammatory biomarker concentrations is also due to higher levels of adiposity that are a likely consequence of UPFD consumption [20], as previous studies have suggested [12]. Although indirect effects varied according to the biomarker examined, our analyses demonstrated adiposity to mediate relationships between UPFD intake and biomarker concentrations, with the percentage of total effect mediated ranging from 12.7 to 70.1% for models including BMI, and 13.5 to 64.5% for models including WHtR. Although once thought to be an energy storehouse, it is now well recognised that adipose tissue has endocrine functions and is metabolically active [22, 51]. Excessive weight gain leads to adipose tissue remodelling with the release of adipose-derived pro-inflammatory cytokines and free fatty acids into circulation, resulting in metabolic dysfunction [52] and increased risk of chronic disease [53]. Previous research by our group, which examined biomarker relationships with modifiable lifestyle factors (smoking, alcohol use, physical activity, dietary quality and adiposity defined by BMI), showed BMI to have the greatest number of significant relationships with inflammatory biomarkers after adjustment for other modifiable factors [54].

Collectively, results from this research suggest that increased intake of UPFDs is associated with a more pro-inflammatory metabolic profile in middle- to older-aged adults, through separate mechanisms, and these may partly underlie reported associations between UPFD consumption and chronic disease risk and mortality. However, the magnitude of effect observed between UPFD weight ratios and inflammatory biomarkers in correlation analyses should be noted, as these suggest weak to moderate strengths of association. Nevertheless, we found that the diets of individuals in our study were quite similar/monotonous [19]. In study populations where eating habits/food culture and preferences are more diverse, greater differences/changes in UPFD consumption could produce stronger associations with inflammatory biomarkers. The examination of UPFD relationships with other chronic non-communicable disease biomarkers may provide further mechanistic insights.

This study has several strengths. As far as we are aware, our research is the first to examine relationships between UPFD consumption, defined using the extensively used NOVA classification, and chronic low-grade inflammation using a wide range of inflammatory biomarkers in a middle- to older-aged population; thus, our study has examined the greatest number of biomarkers in a relatively large population in this context. Research on UPFDs is important for public health in terms of providing better insights into disease causation and informing public health nutrition policy. Ageing is characterised by an increase in the concentration of a number of pro-inflammatory molecules in circulation, a phenomenon that has been termed “inflammageing” [55]. Reduced UPFD consumption may help prevent against systemic inflammation and this may be of particular importance to older adults, who often have low energy requirements and poor diets [56–58]. Other strengths include equal representation by sex (49% male), the use of validated questionnaires to collect data, adjustment for a range of potential confounders, mediation analyses, the use of two measures to define adiposity and further examination of biomarker relationships across UPFD NOVA subgroups.

Despite these strengths, several limitations should be noted. The cross-sectional study design, which precludes drawing conclusions regarding the temporal direction of relationships, limits inference with respect to causality. In addition, the use of self-reported questionnaires is subject to potential inaccuracies, recall and reporting bias. Finally, the generalisability of our findings may be limited. Our data were collected from a single primary care-based sample which may not be representative of the general population. However, Ireland has a generally ethnically homogeneous population [59]. In addition, previous research suggests that approximately 98% of Irish adults are registered with a GP and that, even in the absence of a universal patient registration system, it is possible to perform population-based epidemiological studies that are representative using our methods [60].

## Conclusions

In conclusion, findings from this study demonstrate that higher consumption of UPFDs is associated with a pro-inflammatory biomarker profile in middle- to older-aged adults and that the total effect of UPFD intake on inflammatory biomarker concentrations is likely due both to higher levels of adiposity that are a consequence of UPFD consumption and the inflammatory potential of UPFD products. A more pro-inflammatory profile could partly underlie reported associations between UPFDs and chronic diseases and mortality. Thus, adopting a diet with reduced UPFD intake may help prevent against systemic inflammation, which in turn may reduce chronic disease risk and promote healthy ageing.

## Electronic supplementary material

Below is the link to the electronic supplementary material.

Supplementary Material 1

## Data Availability

The data used and analysed for the purpose of this study are available from the corresponding author on reasonable request.
