# Supplementary material for: Associations between ultra-processed food and drink consumption and biomarkers of chronic low-grade inflammation: exploring the mediating role of adiposity
Source: Eur J Nutr. 2025 Apr 9;64(4):150. doi: 10.1007/s00394-025-03666-1 (PMC11982146; doi:10.1007/s00394-025-03666-1)
Supplement: Supplementary file 1 — Supplementary Material 1 [file 394_2025_3666_MOESM1_ESM.docx]

**Supplementary Table 1. The NOVA food classification.**

| **NOVA group** | **Description** | **Examples of foods and drinks** |
| --- | --- | --- |
| Group1:  Unprocessed  or minimally processed foods and drinks | Foods that have undergone little to no processing and are in their natural state. | Nuts, peanuts and other seeds without salt or sugar; fresh, chilled or frozen meat, poultry, fish and seafood, whole or in the form of steaks, fillets and other cuts; fresh or pasteurised milk; yoghurt without sugar; tea, etc. |
| Group 2:  Processed culinary ingredients | Foods that have been extracted from natural foods or nature by processes such as pressing, grinding, crushing, pulverising and refining. | Oils made from seeds, nuts and fruits, including soybeans, corn, oil palm, sunflower or olives; butter; white, brown and other types of sugar and molasses obtained from cane or beet; lard; honey extracted from the honeycomb; coconut fat; syrup extracted from maple trees; refined or coarse salt, etc. |
| Group 3:  Processed foods and drinks | Foods manufactured by industry with the use of salt, sugar or other substances (Group 2) added to natural or minimally processed foods (Group 1) to preserve or make them more palatable. | Canned or bottled legumes or vegetables preserved in salt (brine) or vinegar or by pickling; canned fish, such as sardines and tuna, with or without added preservatives; tomato extract, pastes or concentrate; salted, dried, smoked or cured meat or fish; bacon; freshly made (unpackaged) bread made of wheat flour; beer, alcoholic cider and wine, etc. |
| Group 4:  Ultra-processed foods and drinks | Foods that have undergone extensive processing and often contain many added ingredients, including preservatives, sweeteners, and artificial flavours or colours. | Fatty, sweet, savoury or salty packaged snacks; pre-prepared meat, fish and vegetables; biscuits, ice creams and frozen desserts; pre-prepared burgers, hot dogs, sausages, chocolates, candies and confectionery in general; cola and other carbonated soft drinks; sauces; packaged bread, desserts and seasonings; sweetened and flavoured yoghurts; breakfast cereals; dairy drinks; sweetened juices; spreads; whisky and gin, etc. |

| **Food subgroups** | **NOVA Group 1** | **NOVA Group 2** | **NOVA Group 3** | **NOVA Group 4** |
| --- | --- | --- | --- | --- |
| Bread |  |  | brown bread,  wholemeal bread | white bread, crackers, pancakes, crisp bread, brown soda bread |
| Cereals | muesli, porridge |  |  | all-bran, bran flakes, cornflakes, sugar-coated cereals |
| Dairy products, fats | eggs | cream, butter, French dressing | cheddar cheese,  low-fat cheddar, brie cheese etc., cottage cheese | dairy desserts, quiche, sunflower margarine, light salad cream, salad cream, other dressings, low-fat margarine,  vegetable oil spread, light butter, olive oil spread,  full-fat yoghurt, low-fat yoghurt |
| Meat, fish, poultry | roast beef, beef steak, minced beef, roast pork, pork chops, pork slices, roast lamb, shellfish, liver, lamb chops, chicken, grilled fish, white fish, oily fish, |  | beef stew, lamb stew, fish roe, pate | beef burgers, ham, sausages, savoury pies, fish-in-batter, fish fingers, fish-in-breadcrumbs, corned beef |
| Potatoes,  rice, pasta | boiled potatoes, roast potatoes, white rice, brown rice, white/green pasta, wholemeal pasta |  | mashed potatoes | chips, potato salad, lasagne, moussaka, pizza, macaroni |
| Soups, sauces spreads |  |  |  | vegetable soup, meat/cream soup, sauces, ketchup, chutney, marmite, jam, marmalade, peanut butter |
| Sweet snacks | peanuts | sugar |  | chocolate biscuits, plain biscuits, crisps, cakes, scones, buns, fruit pies, sweets, sponge puddings, milk puddings, ice cream chocolates, chocolate bars |
| Vegetables | carrots, spinach, broccoli, brussels sprouts, cabbage, peas, green beans, marrow, leeks, cauliflower, parsnips, onions, garlic, mushrooms, sweet peppers, bean sprouts, avocado, dried lentils, cucumber, celery, watercress, tomatoes, sweetcorn, beetroot | green salad,  tofu |  | coleslaw, baked beans |
| Fruits | apples, pears, oranges, grapefruit, bananas, grapes, melon, peaches, strawberries, dried fruit |  | tinned fruit |  |
| Alcoholic drinks |  |  | wine, beer, port | spirits, gin, whiskey |
| Non-alcoholic drinks | tea, coffee, pure juice, full-fat milk, dried milk, low-fat milk, skimmed milk, high-fat/low-fat milk | decaffeinated coffee, buttermilk |  | coffee whitener, cocoa, horlicks, diet soft drinks, soft drinks, fruit squash, soya milk |

**Supplementary Table 2. Composition of food subgroups across the NOVA classification.**

**Supplementary Table 3. Nutritional intake of the study population according to UPFD (weight ratio) intake quartiles.**

| **Variable** | **UPFD weight ratio quartiles (n = 1986)** | | | | |
| --- | --- | --- | --- | --- | --- |
|  | **Q1** | **Q2** | **Q3** | **Q4** | ***p*** |
| **Dietary composition** |  |  |  |  |  |
| Fat, grams/day (mean ± SD) | 59.3 ± 28.4 | 73.3 ± 32.0 | 82.3 ± 34.6 | 94.9 ± 44.7 | <0.001 |
| SFA, grams/day (mean ± SD) | 20.0 ± 10.7 | 25.6 ± 13.1 | 29.2 ± 14.5 | 33.5 ± 16.6 | <0.001 |
| PUFA, grams/day (mean ± SD) | 11.4 ± 6.6 | 14.4 ± 7.5 | 16.6 ± 8.5 | 19.7 ± 12.0 | <0.001 |
| MUFA, grams/day (mean ± SD) | 18.7 ± 9.7 | 23.0 ± 10.3 | 25.8 ± 10.8 | 29.9 ± 14.1 | <0.001 |
| Carbohydrate, grams/day (mean ± SD) | 207.7 ± 100.5 | 242.5 ± 93.9 | 262.4 ± 102.2 | 288.8 ± 123.7 | <0.001 |
| Protein, grams/day (mean ± SD) | 82.4 ± 39.3 | 91.1 ± 34.6 | 93.9 ± 31.3 | 101.1 ± 41.3 | <0.001 |
| Sugar, grams/day (mean ± SD) | 95.2 ± 64.6 | 101.3 ± 50.2 | 109.0 ± 55.8 | 117.3 ± 62.5 | <0.001 |
| Alcohol, milliliter/day (mean ± SD) | 8.8 ± 16.0 | 5.1 ± 10.7 | 4.8 ± 9.3 | 3.3 ± 6.5 | <0.001 |
| Fibre, grams/day (mean ± SD) | 25.2 ± 13.9 | 26.7 ± 11.5 | 26.1 ± 10.9 | 26.5 ± 12.1 | 0.121 |
| **Daily food pyramid shelf servings** |  |  |  |  |  |
| Bread, cereal, potatoes, grains and rice (mean ± SD) | 4.3 ± 2.6 | 5.3 ± 2.9 | 5.6 ± 2.9 | 5.9 ± 3.3 | <0.001 |
| Fruit and vegetables (mean ± SD) | 8.7 ± 7.1 | 7.6 ± 4.7 | 6.6 ± 4.0 | 5.7 ± 3.5 | <0.001 |
| Dairy (mean ± SD) | 1.7 ± 1.4 | 2.0 ± 1.4 | 2.0 ± 1.4 | 2.1 ± 1.5 | <0.001 |
| Meat, fish, poultry and eggs (mean ± SD) | 2.1 ± 1.3 | 2.3 ± 1.2 | 2.4 ± 1.2 | 2.7 ± 1.4 | <0.001 |
| Fats, high fat/sugar foods and drinks (mean ± SD) | 5.2 ± 3.4 | 7.1 ± 4.1 | 9.0 ± 5.0 | 10.3 ± 5.8 | <0.001 |

Abbreviations: MUFA: monounsaturated fatty acids; PUFA: polyunsaturated fatty acids; SFA: saturated fatty acids; UPFD: ultra-processed food and drink.

*p* values determined from a *t*-test and compare Q4 to Q1.

**Supplementary Table 4. Linear regression analysis of the associations between UPFD (weight ratio)**

**intake and inflammatory biomarkers – excluding participants with prevalent diseases.**

| **Biomarker** | **Model 1** | | | **Model 2** | | | |
| --- | --- | --- | --- | --- | --- | --- | --- |
|  | **β** | **95% CI** | ***p*** | **β** | **95% CI** | ***p*** | ***p* (FDR)** |
| C3 | 0.012 | 0.001, 0.023 | **0.03** | 0.014 | 0.002, 0.025 | **0.023** | **0.038** |
| CRP | 0.054 | 0.019, 0.089 | **0.003** | 0.059 | 0.022, 0.097 | **0.002** | **0.006** |
| IL-6 | 0.072 | 0.036, 0.108 | **<0.001** | 0.069 | 0.030, 0.107 | **<0.001** | **0.001** |
| TNF-α | 0.030 | 0.013, 0.046 | **<0.001** | 0.025 | 0.007, 0.043 | **0.006** | **0.013** |
| Adiponectin | -0.017 | -0.046, 0.012 | 0.259 | -0.015 | -0.046, 0.016 | 0.352 | 0.377 |
| Leptin | 0.066 | 0.022, 0.110 | **0.003** | 0.070 | 0.024, 0.117 | **0.003** | **0.008** |
| Resistin | 0.027 | 0.005, 0.049 | **0.015** | 0.029 | 0.005, 0.052 | **0.016** | **0.03** |
| PAI-1 | -0.015 | -0.040, 0.010 | 0.246 | -0.015 | -0.043, 0.012 | 0.277 | 0.32 |
| WBC | 0.019 | 0.006, 0.033 | **0.004** | 0.022 | 0.009, 0.036 | **0.001** | **0.004** |
| Neutrophils | 0.033 | 0.016, 0.050 | **<0.001** | 0.037 | 0.020, 0.054 | **<0.001** | **0.001** |
| Lymphocytes | -0.004 | -0.020, 0.012 | 0.653 | -0.003 | -0.020, 0.014 | 0.711 | 0.711 |
| NLR | 0.037 | 0.017, 0.056 | **<0.001** | 0.040 | 0.019, 0.061 | **<0.001** | **0.001** |
| Monocytes | 0.011 | -0.005, 0.027 | 0.188 | 0.015 | -0.002, 0.031 | 0.085 | 0.116 |
| Eosinophils | 0.024 | -0.007, 0.056 | 0.133 | 0.036 | 0.002, 0.069 | **0.038** | 0.057 |
| Basophils | 0.023 | -0.005, 0.052 | 0.112 | 0.026 | -0.005, 0.057 | 0.1 | 0.125 |

Abbreviations: C3: complement component 3; CRP: c-reactive protein; FDR: false discovery rate; IL-6: interleukin 6; NLR: neutrophil-to-lymphocyte ratio;

PAI-1: plasminogen activator inhibitor 1; TNF-α: tumour necrosis factor-alpha; UPFD: ultra-processed food and drink; WBC: white blood cell count.

Model 1: adjusted for age (in years, continuous) and sex (binary).

Model 2: adjusted for age (in years, continuous), sex (binary), total energy intake (kilocalories, continuous), education (binary), smoking (binary), alcohol use (binary),

physical activity (binary) and anti-inflammatory medication use (binary).

Beta (β) coefficients and 95% confidence intervals (CIs) are shown. Significant *p* shown in **bold**.

**Supplementary Table 5. Linear regression analysis of the associations between UPFD (percentage energy)**

**intake and inflammatory biomarkers.**

| **Biomarker** | **Model 1** | | | **Model 2** | | | |
| --- | --- | --- | --- | --- | --- | --- | --- |
|  | **β** | **95% CI** | ***p*** | **β** | **95% CI** | ***p*** | ***p* (FDR)** |
| C3 | 0.011 | -0.001, 0.022 | 0.069 | 0.008 | -0.003, 0.020 | 0.161 | 0.242 |
| CRP | 0.031 | 0.000, 0.062 | 0.053 | 0.031 | -0.001, 0063 | 0.061 | 0.114 |
| IL-6 | 0.029 | -0.003, 0.062 | 0.077 | 0.033 | 0.000, 0.066 | **0.047** | 0.1 |
| TNF-α | 0.018 | 0.003, 0.034 | **0.021** | 0.016 | 0.000, 0.032 | **0.046** | 0.101 |
| Adiponectin | -0.007 | -0.035, 0.020 | 0.596 | -0.002 | -0.030, 0.025 | 0.858 | 0.858 |
| Leptin | 0.034 | -0.006, 0.073 | 0.095 | 0.026 | -0.014, 0.066 | 0.207 | 0.282 |
| Resistin | 0.030 | 0.011, 0.049 | **0.002** | 0.029 | 0.009, 0.049 | **0.004** | **0.02** |
| PAI-1 | -0.009 | -0.031, 0.014 | 0.447 | -0.007 | -0.029, 0.016 | 0.56 | 0.6 |
| WBC | 0.014 | 0.002, 0.027 | **0.023** | 0.014 | 0.003, 0.026 | **0.017** | 0.064 |
| Neutrophils | 0.027 | 0.012, 0.042 | **0.001** | 0.026 | 0.011, 0.041 | **0.001** | **0.008** |
| Lymphocytes | -0.005 | -0.020, 0.010 | 0.517 | -0.005 | -0.019, 0.010 | 0.536 | 0.6 |
| NLR | 0.032 | 0.014, 0.049 | **<0.001** | 0.031 | 0.013, 0.048 | **0.001** | **0.008** |
| Monocytes | 0.011 | -0.003, 0.025 | 0.12 | 0.016 | 0.002, 0.029 | **0.029** | 0.087 |
| Eosinophils | 0.014 | -0.013, 0.042 | 0.311 | 0.013 | -0.015, 0.042 | 0.346 | 0.433 |
| Basophils | 0.020 | -0.005, 0.046 | 0.112 | 0.022 | -0.004, 0.048 | 0.091 | 0.152 |

Abbreviations: C3: complement component 3; CRP: c-reactive protein; FDR: false discovery rate; IL-6: interleukin 6; NLR: neutrophil-to-lymphocyte ratio;

PAI-1: plasminogen activator inhibitor 1; TNF-α: tumour necrosis factor-alpha; UPFD: ultra-processed food and drink; WBC: white blood cell count.

Model 1: adjusted for age (in years, continuous) and sex (binary).

Model 2: adjusted for age (in years, continuous), sex (binary), education (binary), smoking (binary), alcohol use (binary), physical activity (binary),

anti-inflammatory medication use (binary), type 2 diabetes (binary), cardiovascular disease (binary) and cancer (binary).

Beta (β) coefficients and 95% confidence intervals (CIs) are shown. Significant *p* shown in **bold**.

**Supplementary Table 6. Spearman correlation coefficients between individual UPFD (weight ratio) intake subgroups and inflammatory biomarkers.**

| **Biomarker** | **Bread** | **Cereals** | **Dairy**  **products, fats** | **Meat, fish, poultry** | **Potatoes,**  **pasta** | **Soups, sauces**  **spreads** | **Sweet snacks** | **Vegetables** | **Drinks** |
| --- | --- | --- | --- | --- | --- | --- | --- | --- | --- |
| IL-6, pg/ml | 0.010 | -0.017 | -0.011 | 0.037 | 0.001 | 0.033 | 0.002 | -0.011 | **0.046*** |
| TNF-α, pg/ml | 0.027 | -0.001 | -0.025 | 0.037 | 0.020 | 0.011 | **0.067**** | -0.024 | **0.062**** |
| WBC, 10^9^/l | 0.023 | 0.022 | -0.043 | **0.072**** | -0.013 | **0.059*** | -0.041 | -0.007 | **0.137***** |
| Neutrophils, 10^9^/l | 0.038 | 0.036 | -0.019 | **0.098***** | -0.016 | **0.061**** | -0.021 | 0.013 | **0.147***** |
| NLR | **0.059*** | 0.019 | 0.019 | **0.055*** | -0.011 | 0.033 | 0.018 | 0.034 | 0.030 |
| Basophils, 10^9^/l | -0.014 | 0.023 | -0.002 | 0.042 | 0.033 | 0.034 | -0.007 | 0.013 | **0.055*** |

Abbreviations: IL-6: interleukin 6; NLR: neutrophil-to-lymphocyte ratio; TNF-α: tumour necrosis factor-alpha; WBC: white blood cell count.

Values are presented as Spearman correlation coefficients between UPFD weight ratio subgroups and inflammatory biomarkers among the Mitchelstown Cohort (n = 1986).

Significant correlation coefficient values shown in **bold**.

**p* <0.05; ***p* <0.01; ****p* <0.001.

**Supplementary Figure 1. Participant flow chart.**


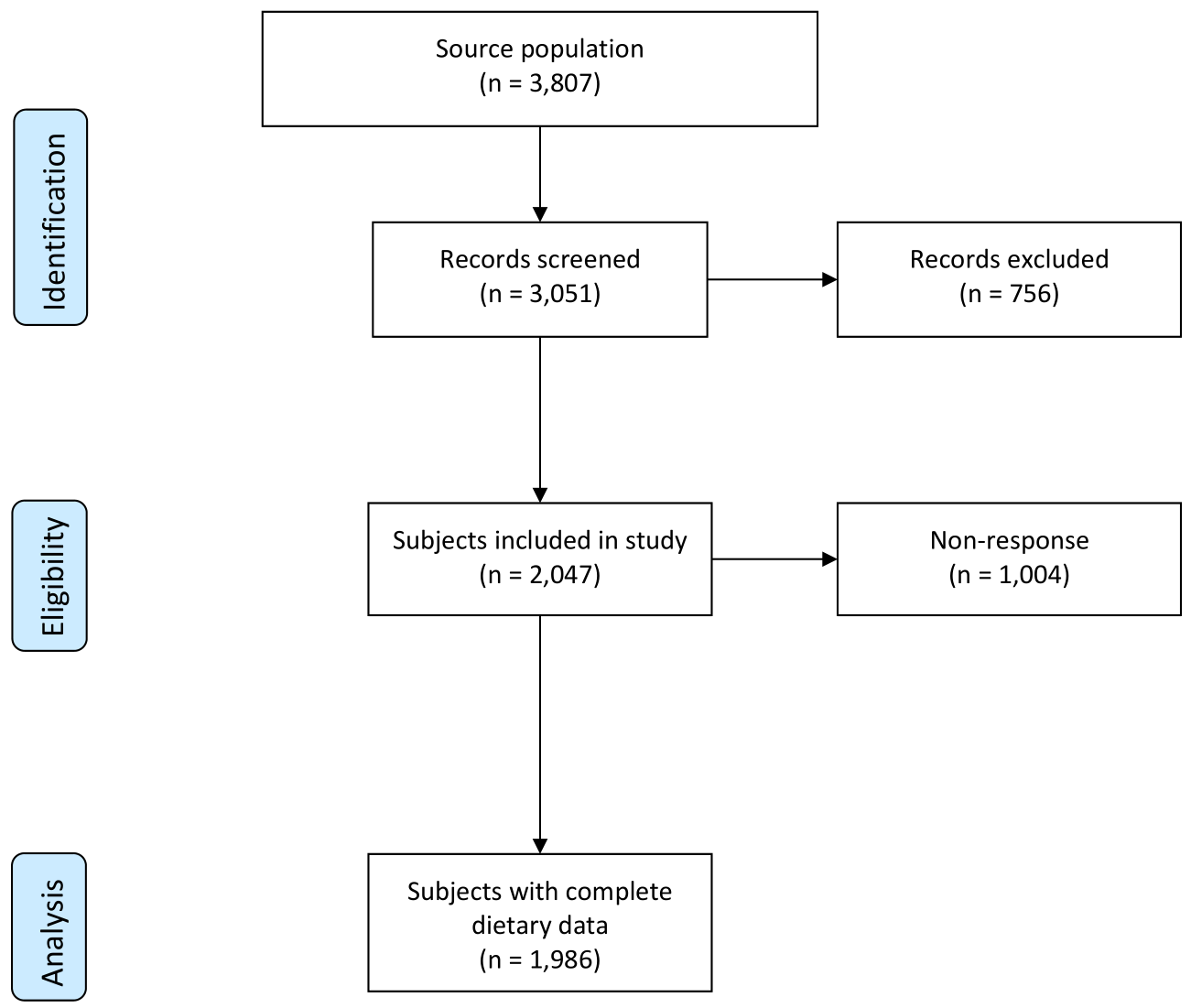


**Supplementary Figure 2. Scatterplot of total UPFD intake (log grams) and total food intake**

**(log grams).**


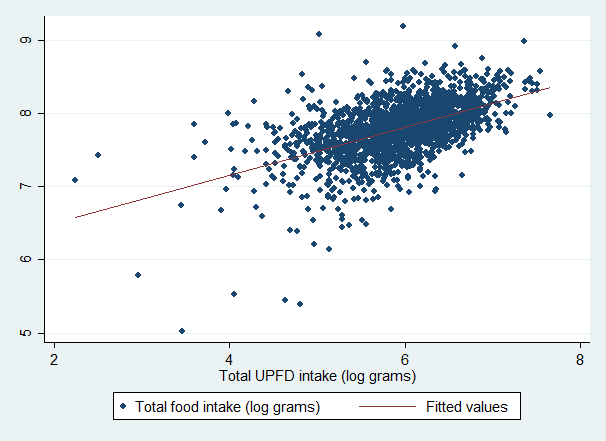


**Figure Legend:** The figure shows a scatterplot of total UPFD intake (log grams) and total food intake (log grams) with a line of best fit. The R^2^ value was 0.285.

**Supplementary Figure 3. Total food intake (log grams) regressed on total UPFD intake (log grams) evaluated by restricted cubic splines.**


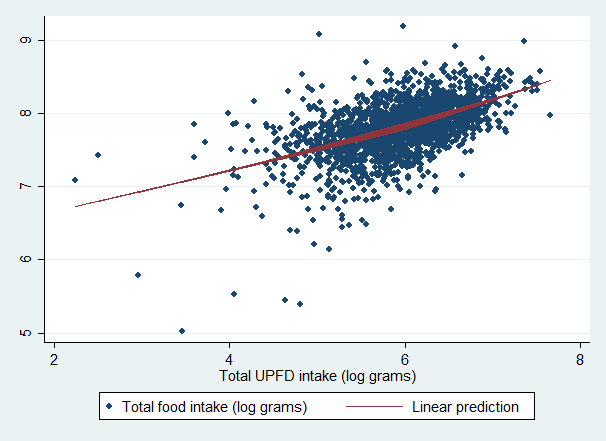


**Figure Legend:** The figure shows total food intake (log grams) regressed on total UPFD intake (log grams) evaluated by restricted cubic splines using five knots. There was no evidence of non-linearity in the restricted cubic spline model (Wald test for linear term: *p* <0.001).
